# Transcriptomic Analysis of Long Non-Coding RNAs and Coding Genes Uncovers a Complex Regulatory Network That Is Involved in Maize Seed Development

**DOI:** 10.3390/genes8100274

**Published:** 2017-10-17

**Authors:** Ming Zhu, Min Zhang, Lijuan Xing, Wenzong Li, Haiyang Jiang, Lei Wang, Miaoyun Xu

**Affiliations:** 1 School of Life Sciences, Anhui Agricultural University, Hefei 230000, China; lyanzhu0@126.com (M.Z.); hyjiang@ahau.edu.cn (H.J.); 2 Biotechnology Research Institute/The National Key Facility for Crop Gene Resources and Genetic Improvement, Chinese Academy of Agricultural Sciences, Beijing 100081, China; 13126532159@163.com (M.Z.); xinglijuan3@163.com (L.X.); lwzm1010@163.com (W.L.)

**Keywords:** maize, seed development, lncRNA, mRNA, co-expression, ceRNA

## Abstract

Long non-coding RNAs (lncRNAs) have been reported to be involved in the development of maize plant. However, few focused on seed development of maize. Here, we identified 753 lncRNA candidates in maize genome from six seed samples. Similar to the mRNAs, lncRNAs showed tissue developmental stage specific and differential expression, indicating their putative role in seed development. Increasing evidence shows that crosstalk among RNAs mediated by shared microRNAs (miRNAs) represents a novel layer of gene regulation, which plays important roles in plant development. Functional roles and regulatory mechanisms of lncRNAs as competing endogenous RNAs (ceRNA) in plants, particularly in maize seed development, are unclear. We combined analyses of consistently altered 17 lncRNAs, 840 mRNAs and known miRNA to genome-wide investigate potential lncRNA-mediated ceRNA based on “ceRNA hypothesis”. The results uncovered seven novel lncRNAs as potential functional ceRNAs. Functional analyses based on their competitive coding-gene partners by Gene Ontology (GO) and KEGG biological pathway demonstrated that combined effects of multiple ceRNAs can have major impacts on general developmental and metabolic processes in maize seed. These findings provided a useful platform for uncovering novel mechanisms of maize seed development and may provide opportunities for the functional characterization of individual lncRNA in future studies.

## 1. Introduction

Maize seed development is initiated by double fertilization. Two male gametes combined to the haploid egg cell and the dikaryotic central cell, respectively, to generate a diploid embryo and a triploid endosperm [1,2]. Mature maize seeds are a composite of maternally derived tissues (pericarp, placenta, and pedicel), endosperm and embryo that provides an exquisite experimental system for developmental analyses. The endosperm accounts for 85% of the kernel mass at maturity, and is of prime agronomic importance. The endosperm function as an absorptive structure that supports embryo development and seedling germination in angiosperms [3]. Typical of grasses, the maize embryo is developmentally precocious. The mature embryo inherits the genetic information for the next plant generation. Recent evidence also indicates that endosperm plays a critical role in the regulation of seed development through interaction with the embryo and the seed coat [4,5]. Differentiation of the endosperm and embryo occurs side by side, within distinct developmental compartments and the embryo and endosperm interact extensively throughout their development. Thus, the maize kernel offers a unique opportunity to study developmental signaling between the embryo, endosperm, and maternal tissues. Elucidation of the genetic regulatory mechanisms involved in maize seed development will facilitate the design of strategies to improve yield and quality.

The transcriptome is the overall set of transcripts. Analysis of transcriptome dynamics can be used to determine aids the function of unannotated genes, identify genes that act as critical network hubs, and interpret the cellular processes associated with development. In maize, high-throughput RNA sequencing (RNA-seq) has provided insights into the programs controlling the development of different organ systems including leaves, shoot apical meristems, and the endosperm, among others, as well as the general development of whole seed [6,7,8,9]. Furthermore, a study that took a global view of transcriptome dynamics over the majority of seed development stages has been reported [10]. In another study, coupled laser-capture microdissection (LCM) and RNA-Seq were used to comprehensively identify the mRNA populations present in each of the main maize endosperm cell types, as well as the embryo and four maternal compartments of the kernel at 8 days after pollination (DAP). Specifically accumulated mRNAs in each of the compartments and co-expressed gene modules were detected [11]. The above efforts have been made on studying the role of mRNA and the gene regulatory networks during seed development in maize. However, seed development is a tightly regulated process, which requires exquisite control over gene expression. Thus, additional studies that utilize recent advancements in biology are required.

Non-coding RNAs have recently emerged as versatile master regulators of biological functions. Long non-coding RNAs (lncRNAs) are a large and diverse class of transcribed ncRNAs wih lengths ranging from 200 nt to 100 kb. They play an important role in the regulation of gene expression, and act by acting as competing endogenous RNAs (ceRNAs) [12,13,14]. Plant lncRNAs are transcribed by different RNA polymerases and have diverse structural features. They are also important regulators of gene expression in various biological processes [15]. Their regulation occurs through a large complex network that involves mRNAs, micro RNAs (miRNAs), and proteins in animals [16], and they have multi-faceted biological functions that vary based on location, binding site, and mode of action. Recently, lincRNAs (long intergenic noncoding RNAs) have also been shown to function as miRNA targets or decoys in plants [17]. However, their main functions remain unclear. miRNAs are small endogenous ncRNAs of 18–24 nucleotides in length that originate from long self-complementary precursors. Besides their direct involvement in developmental processes, plant miRNAs play key roles in gene regulatory networks and various biological processes. In addition to the conventional miRNA function, a reversed miRNA logic exists, in which coding and noncoding RNA targets can crosstalk through their ability to compete for miRNA binding [18]. On the basis of this hypothesis, ceRNAs have recently been discovered, adding to the complexity of miRNA-mediated gene regulation [19]. CeRNAs are RNAs that share miRNA recognition elements (MREs), thereby competing for miRNA binding sites and regulating each other. Several studies that analyzed lots of reports of tissues and mammalian cells have shown that the combined effects of multiple ceRNAs can have a major impact on gene expression and cellular phenotypes [20,21,22]. However, few ceRNA interactions have been found in plants.

LncRNAs acting as potential ceRNAs can compete for the same MREs and regulate protein expression. CeRNAs are implicated in the development of some cancers. A disruption in the delicate ceRNA network can lead to tumor formation [23]. However, no studies on ceRNA involvement in plant development have been published to date. Understanding miRNA mediated lncRNA and mRNA crosstalk can provide significant insight into gene regulatory networks and their implications for seed development.

Here, we performed high-throughput sequencing analysis to determine the expression profiles of lncRNAs and mRNAs during embryo and endosperm differentiation stages. We systematically identified novel and seed-specific lncRNAs, and then the differential expressions of representative lncRNAs were further confirmed using quantitative real-time polymerase chain reaction (qRT-RCR). The potential function of lncRNAs and their target genes were also predicted and analyzed. Subsequently, we determined the comprehensive functional landscape of the lncRNA-miRNA-mRNA ceRNA networks in maize seed development for the first time using bioinformatics approaches, and acquired mRNA associated pathways and gene ontology data. The result provides new insights into the regulatory mechanism of lncRNAs in seed development.

## 2. Materials and Methods

### 2.1. Plant Material and RNA Isolation

The maize (*Zea mays*) inbred line B73 was grown in the field in the summer of 2015 in Langfang, Heibei, China. Ears were self-pollinated. Embryos and endosperm were collected and dissected at 9DAP, 15DAP, and 20DAP, frozen immediately in liquid nitrogen, and stored at −80 °C before processing. The samples were obtained from at least three plants. Total RNA was extracted using TRIzol reagent (Invitrogen, Carlsbad, CA, USA). Total RNA concentration and quality were measured using the NanoDrop system (Thermo Fisher Scientific Inc., Waltham, MA, USA). All of the sequenced samples were generated from high-quality RNA samples with having both 28S/18S > 1 and A260/A280 is between 1.8 and 2.1.

### 2.2. Library Construction, RNA-Sequencing and Data Sets Used for the Identification of lncRNAs

Strand-specific RNA-seq (ssRNA-seq) libraries were prepared according the manufacturer’s instructions using the Illumina Standard RNA sample library preparation kit (Illumina, San Diego, CA, USA). After quantification using the Agilent 2100 bioanalyzer (Agilent, Santa Clara, CA, USA), the strand-specific libraries were sequenced on an Illumina HiSeq 2500 instrument that generated paired-end reads of 100 nucleotides. Library construction and Illumina sequencing were performed by OE Biotech CO., LTD (Shanghai, China). A total of 12 high throughput RNA seq data were surveyed for the identification of lncRNAs and mRNAs in maize seed. The data have been submitted to the NCBI SRA database [24] with accession numbers SAMN07508173, SAMN07508174, SAMN07508175, SAMN07508176, SAMN07508177, and SAMN07508178.

### 2.3. Bioinformatics Identification of Maize Seed lncRNAs

**Figure 1:**
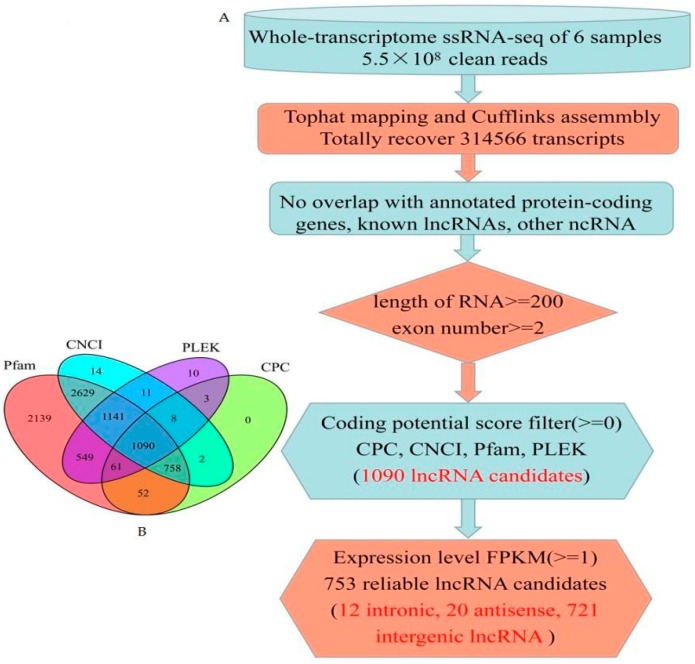
Informatics pipeline for the identification of maize lncRNAs. (**A**) Schematic diagram of the informatics pipeline; (**B**) Venn diagram showing the numbers of filtered potential lncRNAs by Coding Potential Calculator (CPC), Coding-Non-Coding index (CNCI), the protein family database (Pfam), and predictor of long non-coding RNAs and messenger RNAs based on an improved k-mer scheme (PLEK).

The pipeline used for the identification of lncRNA has been described in Figure 1. The RNA sequence data were quality evaluated and filtered using FASTQC [25] and NGS QC TOOLKIT v2.3.3 [26]. Cleaned reads were aligned to the *Zea mays* reference genome using Tophat 2.0 program [27]. After the alignment, Cufflinks [28] was employed to assemble reads into transcripts according to the instructions provided. The assembled transcripts were selected for further analysis. The number of fragments per kilobase per million mapped reads (FPKM) per gene was calculated [29]. Next, we discarded transcripts that overlapped with known protein-coding genes on the same strand, transcripts with FPKM scores <1, transcripts shorter than 200 nt, and an exon number of less than 2. We used the Coding Potential Calculator (CPC) [30], Coding-Non-Coding index (CNCI) [31], and predictor of long non-coding RNAs and messenger RNAs based on an improved k-mer scheme (PLEK) [32] to filter transcripts with coding potential. The remaining transcripts with known protein domains were excluded by Pfam Scan according to Pfam HMM [33]. The transcripts that remained were considered reliably expressed lncRNAs. lncRNAs were classified into intergenic, intronic, antisense, and sense lncRNAs using the cuffcompare program in the Cufflinks suite [34]. Significant differently expressed (DE) lncRNAs between embryo and endosperm were extracted. The change of lncRNA expression was calculated as the fold change (FC) = FPKM of endosperm/FPKM of embryo. Only the lncRNAs that met the criteria of log2FC ≥1 or ≤−1 with *p*-value < 0.05 were considered to be DE lncRNAs.

### 2.4. Expression Analysis

Differentially expressed lncRNA and mRNA were screened for a *p*-value less than 0.05 and fold change in more than 2.0. Then, difference integration analyses (Venn analyses) were performed. The often characteristic elements of each stage were determined by Venn analysis. The up and downregulated RNAs were illustrated using pie graphs and different colors. DE lncRNAs, and mRNAs were analyzed using Cluster software v3.0 (Human Genome Center, University of Tokyo, Tokyo, Japan). Normalized expression levels of each RNA type were further analyzed with hierarchical clustering HCL. The results were presented using Hierarchical Clustering Explorer 3.5 [35].

### 2.5. Quantitative Real Time PCR (qRT-PCR) Validation of lncRNAs

Total RNA was isolated, respectively, from maize embryo and endosperm for qRT-PCR using TRIzol reagent (Invitrogen, Carlsbad, CA, USA). First-strand cDNA was reverse transcribed by PrimeScriptTM RT reagent Kit (TakaRa Biotechnology Co., Dalian, China). The qRT-PCR was performed using SYBR Premix Ex TaqTM (TakaRa Biotechnology Co., Dalian, China). The ACTIN was used as the reference gene and all of the primers used were as listed in File S1. Quantification of lncRNA expression was performed using the comparative CT method, and the specificity of the amplified product was evaluated by melting curve. This experiment was performed by three technical replicates and three biological replicates. 

### 2.6. Construction and Analysis of the lncRNA-miRNA-mRNA ceRNA network

A total of 158 known miRNA sequences of *Zea mays* [36] were utilized for interaction analyses with lncRNA and mRNA sequences. To find the potential deregulated lncRNA during the developmental process, the lncRNA-miRNA-mRNA ceRNA network for seed development was constructed based on the “ceRNA hypothesis” as follows: first, the correlation between DE mRNAs and DE lncRNAs was evaluated using the Pearson correlation coefficient (PCC) from matched mRNA and lncRNA expression profile data. The lncRNA-mRNA pairs with PCC > 0.5 and *p* < 0.01 were selected as co-regulated lncRNA-mRNA pairs. Then, the PCC between miRNAs and DE mRNAs, between miRNAs and DE lncRNAs, were computed from paired miRNA, mRNA, and lncRNA expression profile data. For a given lncRNA-mRNA pair, both mRNAs and lncRNAs in this pair are targeted and negatively co-expressed with a certain common miRNA to identify interaction was identified as a competing triplet. We generated the ceRNA score of an lncRNA-mRNA pair targeted by common miRNAs to measure the likelihood of an lncRNA acting as a ceRNA to a protein coding gene. The ceRNA score was calculated the number of MREs for the distinct shared miRNAs between the pair as opposed to the total number of MREs for all distinct miRNAs targeting the lncRNA. Then, we calculated the *p*-value for each potential ceRNA pair using a hypergeometric test that considered the number of shared miRNAs between a ceRNA pair against the number of miRNAs targeting individual components of the ceRNA pair [37,38].

## 3. Results

### 3.1. Genome-Wide Identification of lncRNAs in Maize Seed

Total RNA was extracted from maize embryos and endosperm of maize at 9, 15, and 20 DAP. High-throughput Illumina paired-end sequencing technology was used to profile the maize lncRNAs and protein-coding transcripts. In total, 573 million raw reads were generated from six samples. After trimming adapters and filtering out low quality reads, approximately 553 million clean reads with 87.71–89.25% mapping to the *Zea mays* genome were obtained and used for further analysis. The details of the RNA-seq data are shown in File S2.

To characterize maize seed lncRNAs, we developed a computational identification pipeline based on whole transcriptome ssRNA-seq data (Figure 1). A total of 314,566 transcripts were reconstructed from all of the six RNA-seq datasets using cufflink 2.0 [28]. Three filter processes descripted in method were applied to distinguish lncRNAs from protein-coding transcript units. We obtained 753 reliably expressed lncRNAs, consisting of 12 intronic lncRNAs, 20 antisense lncRNAs, and 721 intergenic lncRNAs (File S3).

### 3.2. Expression Profiles of lncRNAs during Embryo and Endosperm Development

**Figure 2:**
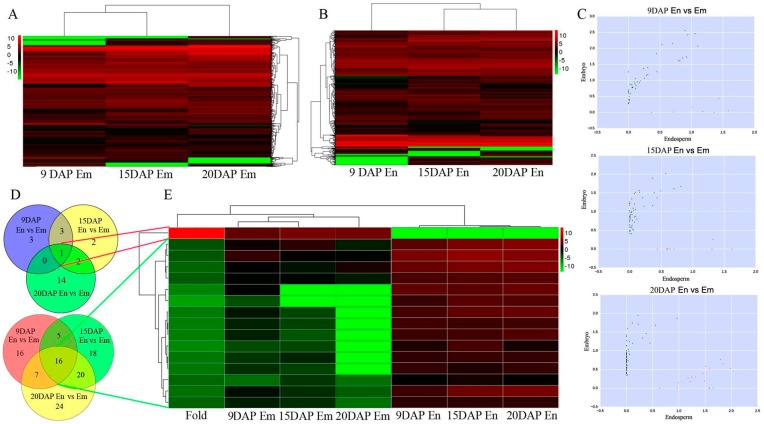
Expression profiles of lncRNAs during embryo and endosperm development. (**A**) Cluster heat map of all lncRNAs expression at different stages of embryo development; (**B**) Cluster heat map of all lncRNAs expression at different stages of endosperm development; (**C**) Scatter plots showing differentially expressed lncRNAs in the embryo (black points) and endosperm (red points) at different stages; (**D**) Differentially expressed lncRNAs between embryo and endosperm at different stages; (**E**) Hierarchical clustering showing often up- and downregulated lncRNAs in the two tissues.

In total, 512 and 614 pair-mapped lncRNAs (FPKM > 1 in at least one library) from embryo and endosperm were genomic-wide systematically detected and the whole expression profile is presented in Figure 2A,B and File S4. LncRNA transcripts length ranged from 202 to 8253 nucleotides, with a mean of 1261 nucleotides (Figure S1). The lncRNAs showed fewer average counts (FPKM = 4.44) than the coding transcripts (FPKM = 11.69) (Figure S2).

Three comparison groups were assigned based on the different stages of seed differentiation, endosperm vs. embryo (9 DAP), endosperm vs. embryo (15 DAP), and endosperm vs. embryo (20 DAP) (Figure 2C). For 9 DAP seeds, 3 lncRNAs were upregulated, and 16 lncRNAs were downregulated (Figure 2D; Table 1). For 15 DAP seeds, 2 lncRNAs were upregulated, and 18 lncRNAs were downregulated (Figure 2D; Table 1). For 20 DAP seeds, 14 lncRNAs and 24 lncRNAs were upregulated and downregulated, respectively (Figure 2D; Table 1). All of the DE lncRNAs were statistically significant (*p*  <  0.05) with fold changes greater than 2.0. VENN analysis revealed that 1 lncRNA were upregulated and 16 lncRNAs were downregulated at all three stages of seed development (Figure 2D; Table 1). Clusters for RNA expression were generated and analyzed using hierarchical clustering (HCL) for 1 lncRNA and 16 lncRNAs that were up-or downregulated in all stages (Figure 2E, Files S5 and S6). Of the lncRNAs, 97.52% were intergenic, 1.98% were located in an intron, and 0.495% were anti-sense (File S3). These results differ from findings based on other models [16,39], suggesting that lncRNA expression differs among different tissues and systems. In addition, 135 and 237 lncRNAs were specifically expressed in the embryo and endosperm, respectively (Figure S3A). Only 1 lncRNA was expressed exclusively in the embryo (log2FC >1 or < – 0.5, and *p* < 0.05) (File S5; Figure S3B,C), whereas 13 endosperm-specific lncRNAs were expressed in endosperm at all three stages (Figure S3D,E). Sixteen lncRNAs exhibited a >10-fold increase in the endosperm compared to the embryo (File S6; Figure S3D,E).

### 3.3. Expression Profiles of mRNAs during Embryo and Endosperm Development

**Figure 3:**
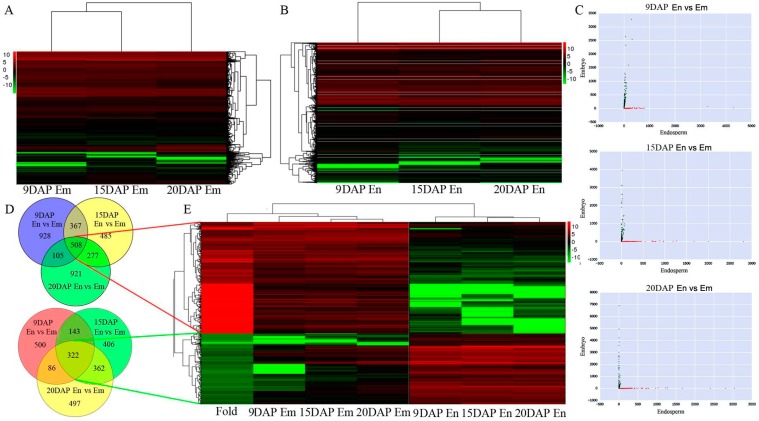
Expression of mRNA during embryo and endosperm development; (**A**) Cluster heat map of mRNA expression at different stages of embryo development; (**B**) Cluster heat map of mRNA expression at different stages of endosperm development; (**C**) Scatter plots showing differentially expressed mRNAs between embryo (black points) and endosperm (red points) at different stages; (**D**) Differentially expressed mRNAs between embryo and endosperm at different stages; (**E**) Hierarchical clustering showing up- and downregulated mRNAs in the two tissues.

41,692 and 37,449 mRNAs (FPKM > 1 in at least library) were detected in the embryo and endosperm, respectively, (Files S7 and S8); the whole expression profile is shown Figure 3. Similar to lncRNA, three comparison groups were assigned based on the different stages of seed differentiation, endosperm vs. embryo (9 DAP), endosperm vs embryo (15 DAP), and endosperm vs. embryo (20 DAP) (Figure 3C). For 9 DAP seeds, 928 mRNAs were up regulated and 500 mRNAs were downregulated (Figure 3D; Table 1). For 15 DAP seeds, 1485 mRNAs were upregulated and 406 mRNAs were downregulated (Figure 3D; Table 1). For 20 DAP seeds, 921 mRNAs were upregulated and 497 mRNAs were downregulated (Figure 3D; Table 1). All of the DE mRNAs were statistically significant (*p*  <  0.05) with fold changes greater than 2.0. VENN analysis revealed that 508 mRNAs were upregulated and 322 mRNAs were downregulated at all three stages of seed development (Figure 3D; Table 1). Clusters were generated and analyzed with HCL for the often differentially regulated 508 mRNAs and 322 mRNAs that were up-or downregulated (Figure 3E).

### 3.4. Validation of lncRNA Expression Using qRT-PCR

**Figure 4:**
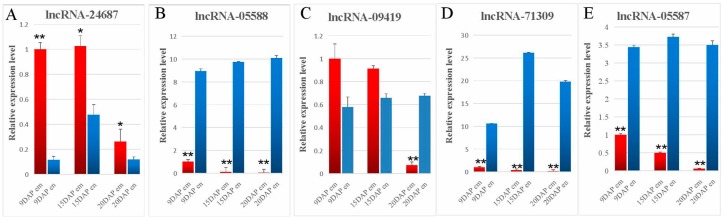
Validation of the differences in lncRNAs. To validate the differentially expressed lncRNAs identified by the RNA-seq results. qPCR was performed to test the expressions of one embryo-specific upregulated lncRNA, and four downregulated lncRNAs. Red bars and blue bars stand for tissues came from embryo and endosperm, respectively. The relative expression of the five differentially regulated lncRNAs normalized to ACTIN. The data in the figures represent the averages ±SD. * *p* < 0.05, ** *p* < 0.01, and *** *p* < 0.001 based on one-way ANOVA.

To confirm the embryo- and endosperm-specific expressions, five lncRNAs were selected for qRT-PCR, including one embryo-specific and four endosperm-specific lncRNAs. As shown in Figure 4, qRT-PCR and the RNA-seq data showed similar lncRNA expression patterns, despite some differences in expression level. For example, although both RNA-seq and qRT-PCR showed that this lncRNA-24687 was upregulated in embryos, qRT-PCR revealed that this lncRNA was expressed in both in the embryo and endosperm and its expression decreased at 20 DAP (Figure 4A). LncRNA-09419 was also expressed in both the embryo and endosperm (Figure 4C).

### 3.5. Construction and Analysis of the lncRNA-miRNA-mRNA ceRNA Network

**Figure 5:**
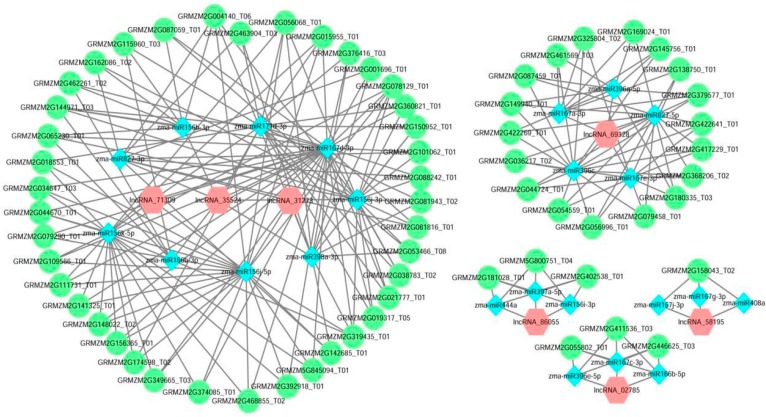
Co-expression network of lncRNA-miRNA-mRNA and ceRNA. The network was constructed based on ceRNA score (score ≥ 1, *p*-value < 0.01). Diamond nodes represent miRNAs, circles represent mRNAs, and rectangles represent lncRNAs.

Some lncRNAs play important roles in the regulation of gene expression by acting as ceRNAs, thereby indicating an additional layer of complexity [12]. To investigate the possible functions of maize seed lncRNAs, we predicted the potential lncRNA as ceRNA, according to the “ceRNA hypothesis”, ceRNA members can compete for the same MREs to regulate each other. RNA transcripts communicate through the ceRNA language [18]. Based on our analyses, we constructed lncRNA-miRNA-mRNA co-expression networks. There were 236 edges, and 5 miRNAs, 7 lncRNAs, and 69 mRNAs were included in the networks (Figure 5). As showed in Figure 5, a large proportion of mRNAs communicated with individual lncRNAs, and all lncRNAs acted as ceRNAs to communicate with multiple mRNAs by competing for specific shared miRNAs. These results suggest that the aberrant expression of lncRNA ceRNA would result in the extensive variation in gene expression through miRNA-mediated lncRNA-mRNA ceRNA crosstalk interactions, implying that ceRNA are important for lncRNA function during seed development. Three lncRNAs (lncRNA_35524, lncRNA_31273, and lncRNA_69328 were only expressed in embryo, and four (lncRNA_71309, lncRNA_02785, lncRNA_86055, and lncRNA_58195) were expressed specifically in endosperm (Table 2). These seven lncRNAs with ceRNA activity are potentially associated with seed development.

**Figure 6:**
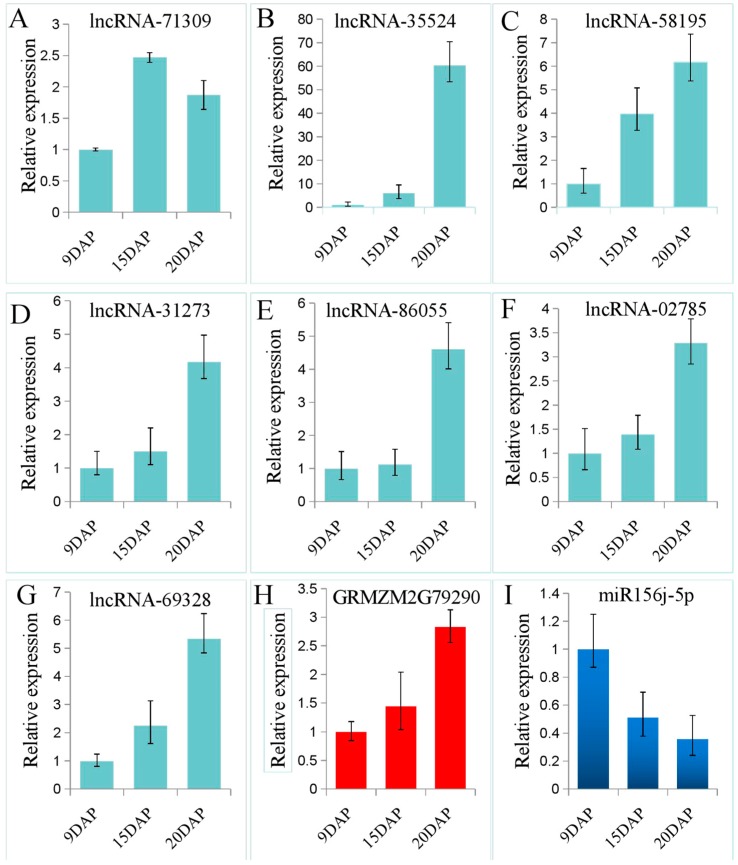
Validation of the expression profiles of seven lncRNAs, GRMZM2G79290 and miR159j-5p in endosperm at 9 DAP, 15 DAP, and 20 DAP. To validate the seven lncRNAs can potentially regulate mRNA as ceRNA trough miRNA, GRMZM2G79290, and miR159j-5p were selected to perform qPCR to test the expressions of lncRNA deregulated mRNA. Blue bars stand for lncRNAs, red bars and dark blue bars stand for mRNA and miRNA, respectivelly. The relative expression of the seven lncRNAs and one mRNA normalized to ACTIN. The relative expression of miRNA normalized to U6.

To confirm the expression profiles of the seven lncRNAs, endosperm tissues of 9DAP, 15DAP, and 20 DAP were selected for qRT-PCR. As shown in Figure 6, the results of qRT-PCR were a little different with the RNA-seq data. lncRNA_35524, lncRNA_31273, and lncRNA_69328 were expressed in endosperm (Figure 6B,D,G). Except for lncRNA_71309, the expression levels of lncRNA_35524, lncRNA_58195, lncRNA_31273, lncRNA_86055, lncRNA_02785 and lncRNA_69328 were increased with kernel development (Figure 6A–G).

To valid ceRNA potentiality of the above seven lncRNAs, we selected GRMZM2G79290 and miR159j-5p according to Figure 5 to perform qRT-PCR. The result showed that expression profile of GRMZM2G79290 was similar to the above six lncRNAs, but opposite to miRNA’s (Figure 6H,I).

**Figure 7:**
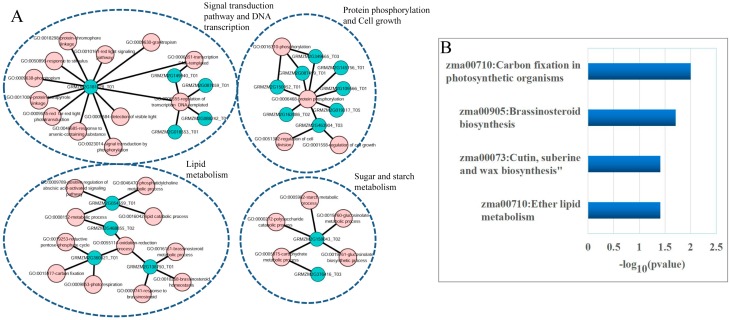
Functional analysis of the deregulated lncRNAs. (**A**) Functional enrichment map of gene ontology (GO) terms for mRNAs as ceRNA counterparts of lncRNAs. Each pink node represents a GO term and each blue node represents the proportion of shared genes between connecting GO terms; (**B**) The enriched Kyoto Encyclopedia of Genes (KEGG) pathways ranked by –log_10_ (*p* value).

To investigate the potential functional implication of these seven lncRNAs, we performed a functional enrichment analysis of gene ontology (GO) and Kyoto Encyclopedia of Genes and Genomes (KEGG) for mRNAs in the lncRNA-associated ceRNA network. GO analysis revealed 39 enriched GO terms in the Biological Process category (*p* < 0.05 and Fold Enrichment > 4.0) (File S9), which could be clustered into four functional sub-networks involved in signal transduction and DNA transcription, protein phosphorylation and cell growth, lipid metabolism, and sugar and starch metabolism (Figure 7A). KEGG analysis focusing on the biological pathways showed that these mRNAs that acted as ceRNA and interacted with lncRNAs in maize kernels, were significantly enriched in several pathways involved in carbon fixation, brassinosteroid biosynthesis, cutin, suberine and wax biosynthesis, and ether lipid metabolism (*p* < 0.05 and Fold Enrichment > 4.0) (Figure 7B and File S9). These enriched biological processes and pathways have been reported to play important roles in seed development. For example, suberin is an important component of seed coats and brassinosteroids are important signaling molecules in seed development [40].

## 4. Discussion

The conventional view of gene regulation focused on protein-coding genes until the discovery of numerous non-coding RNAs including lncRNAs and miRNA. A substantial number of lncRNAs exist in mammals and plants, and they play important functional roles in human disease, plant development, and other biological processes [41,42]. However, a comprehensive analysis of lncRNA expression in maize seed development has not yet been performed until now. 9 DAP, 15DAP, and 20DAP represented three typical time-points in maize kernel development [43]. The endosperm already completed differentiation, with the aleurone, transfer cell and starchy endosperm cells at 9 DAP [44]. Additional cell types start to differentiate in the embryo at 9 DAP [45]. At around 15 DAP, embryo-surrounding region disappears together with the suspensor and synthesis of endosperm starch and storage proteins reach peaks [43,45]. Programmed cell death occurs from 20 DAP [43]. This study is the first report on the expression of lncRNA during the different developmental stage of embryos and endosperm in maize. To date, systematic searches for lncRNAs have been conducted in 13 maize tissues, including 25 DAP embryo and 25 DAP endosperm [46]. In this study, we identified 753 reliably expressed lncRNAs and found that they share similar features with those identified in the other 13 previously tested maize tissues. LncRNAs are shorter and are expressed at significantly lower levels than protein-coding transcripts [41]. In addition, many lncRNAs are expressed in a tissue-specific manner, suggesting that lncRNAs expression is biologically and evolutionally controlled.

MiRNAs mediate communication between transcripts during the development of different tissues through MREs. In this study, we constructed a ceRNA network to predict the function of lncRNAs. The lncRNAs and mRNAs in ceRNA network exhibited dynamic expression and regulation patterns during the developmental processes, suggesting that ceRNA interactions also mediate the coordination of different functions during seed development.

From the lncRNA-miRNA-mRNA co-expression network, we found a total of 23 miRNAs belonging to 9 miRNA families were co-expressed with 7 lncRNAs and 69 mRNAs, forming five complex networks (Figure 5). The nine families comprised miR156, miR166, miR167, miR171, miR396, miR398, miR408, miR444, and miR827. The miR156/SPL module is highly conserved among the phylogenetically distinct plant species, and plays important roles in regulating plant fitness, biomass, and yield [47]. Osmotic stress reduced miR167a, which targets IAR3, then the miR167/ARF module affects auxin conjugation to coordinate growth and patterning in plants [48]. MiR171 is involved in GA and auxin homeostasis by targeting GRAS family members in tomato [49]. The miR396c-OsGRF4-OsGIF1 regulatory module plays an important role in grain size determination and has implications for rice yield improvement [50]. MiR444a plays multiple roles in the rice NO3-signaling pathway that affects nitrate-dependent root growth, nitrate accumulation, and phosphate-starvation responses [51]. The miR827/NLA module plays an important role in phosphate transport activity [52]. In addition to protein and second messengers, small regulatory RNAs also play a role in signal transduction. In this study, we investigated the role of ncRNAs regulation in seed development. GO analyses were performed to further annotate the biological functions of ceRNAs in the lncRNA-ceRNA network. We noticed that a significant number of GO terms were related to signal transduction (Figure 6A). This phenomenon is very interesting for the important roles of co-expressed miRNAs and their target genes in seed development. However, no combination of lncRNAs and seed development has been made before.

In summary, the application of ceRNA network analysis to transcriptomes obtained during tissue development provides a novel approach for understanding gene functionality, and give us new insights on non-coding RNA regulatory in seed development.

## Figures and Tables

**Table 1:** Differentially expressed long non-coding RNAs (lncRNAs) and mRNAs in embryos and endosperm.

RNAs	stages	Upregulated	Often Upregulated	Downregulated
lncRNAs	9 DAP	7	1	44
	15 DAP	8		59
	20 DAP	17		67
mRNAs	9 DAP	1908		1052
	15 DAP	1637	508	1233
	20 DAP	1811		1267

**Table 2:** Detailed information of seven lncRNAs with ceRNA activity during the seed development.

ID	Genomic Location	False Discovery Rate (FDR) (9 DAP Em/En)	FDR (15 DAP Em/En)	FDR (20 DAP Em/En)
lncRNA_35524	Chr1: 55243404-55249539	1.11	2.92	8.23
	55250972-55251328			
lncRNA_58195	Chr6: 14305962-14306059	0.1	0.01	0.01
	14311356-14312539			
lncRNA_71309	Chr7: 135515079-135515358	0.008	0/495	0/375
	135515534-135515693			
	135520571-135520702			
	135520779-135521159			
lncRNA_31273	Chr3: 118012172-118012521	1.56	6.54	24
	118013700-118014996			
lncRNA_86055	Chr1: 137900175-137900970	0/64.5	0/62.9	0/21.2
	137901054-137901119			
lncRNA_02785	Chr1: 134602111-134603048	0.08	0.06	0.004
	134603153-134603273			
lncRNA_69328	Chr7: 3875642-3879270	15.2	6	4
	3879378-3879427			

## Supplementary Materials

Supplementary materials can be found at www.mdpi.com/2073-4425/8/10/274/s1. File S1: List of primers used in this study; File S2: Summary of *Zea mays* RNA-seq data; File S3: Characteristics of all lncRNA identified in this study; File S4: Characteristics of lncRNA identified in embryo and endosperm; File S5: Up-regulated lncRNAs at three stages (endosperm vs embryo); File S6: Down-regulated lncRNAs at three stages (endosperm vs embryo ); File S7: Characteristics of mRNA identified in embryo; File S8: Characteristics of mRNA identified in endosperm; File S9: GO analysis for mRNAs in the lncRNA-associated ceRNA network; Figure S1: lncRNA length distribution; Figure S2: Expression of lncRNAs and mRNA transcripts; Figure S3: Expression patterns of embryo or endosperm specific LncRNAs.
