# Supplementary figures and images for: Transcriptomic Analysis of Long Non-Coding RNAs and Coding Genes Uncovers a Complex Regulatory Network That Is Involved in Maize Seed Development
Source: Genes (Basel). 2017 Oct 17;8(10):274. doi: 10.3390/genes8100274 (PMC5664124; doi:10.3390/genes8100274)

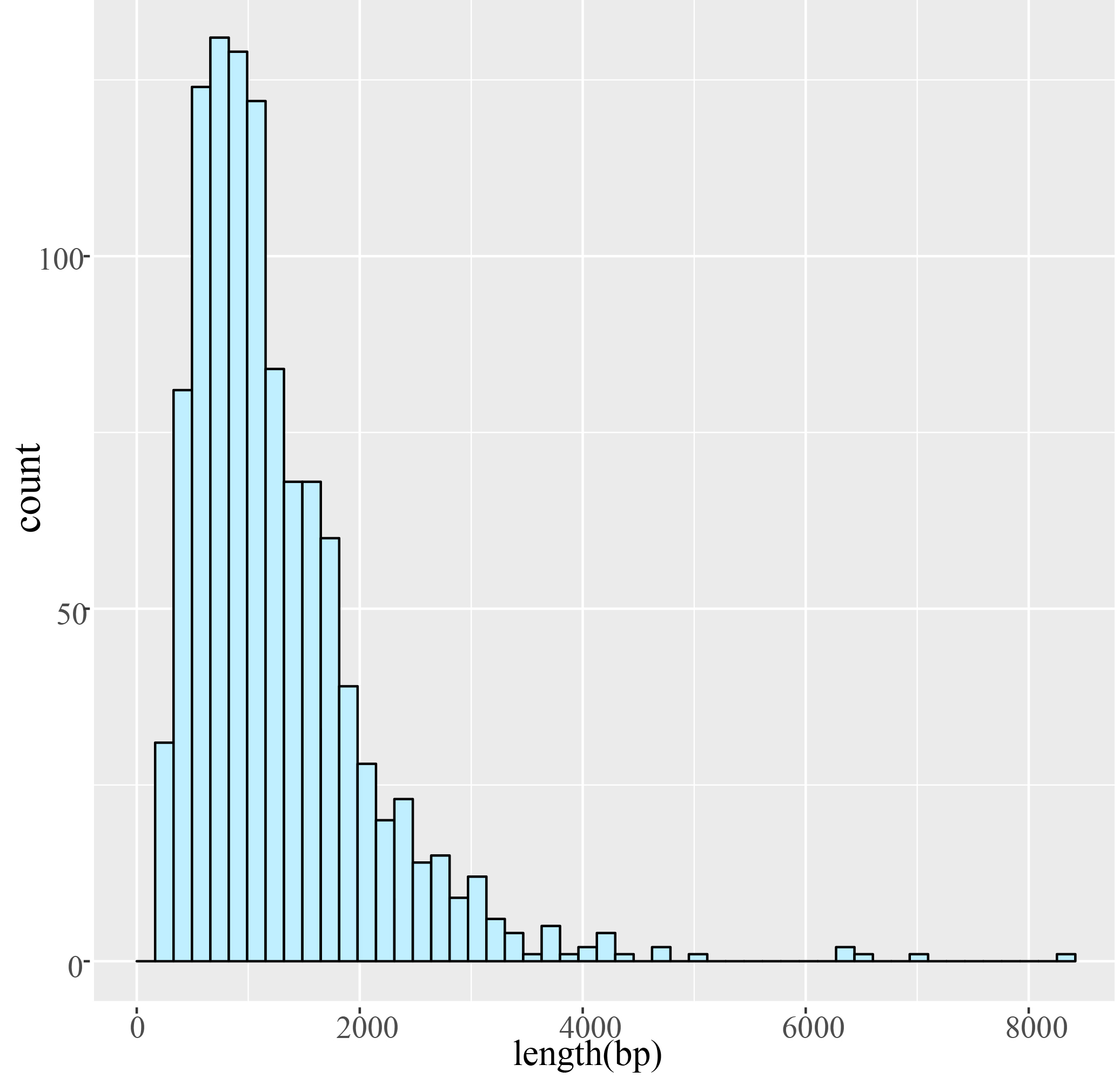

Supplement: Supplementary file 1 [file genes-08-00274-s001.zip › Supplementary files/Figure S1 lncRNA length distribution.jpg]

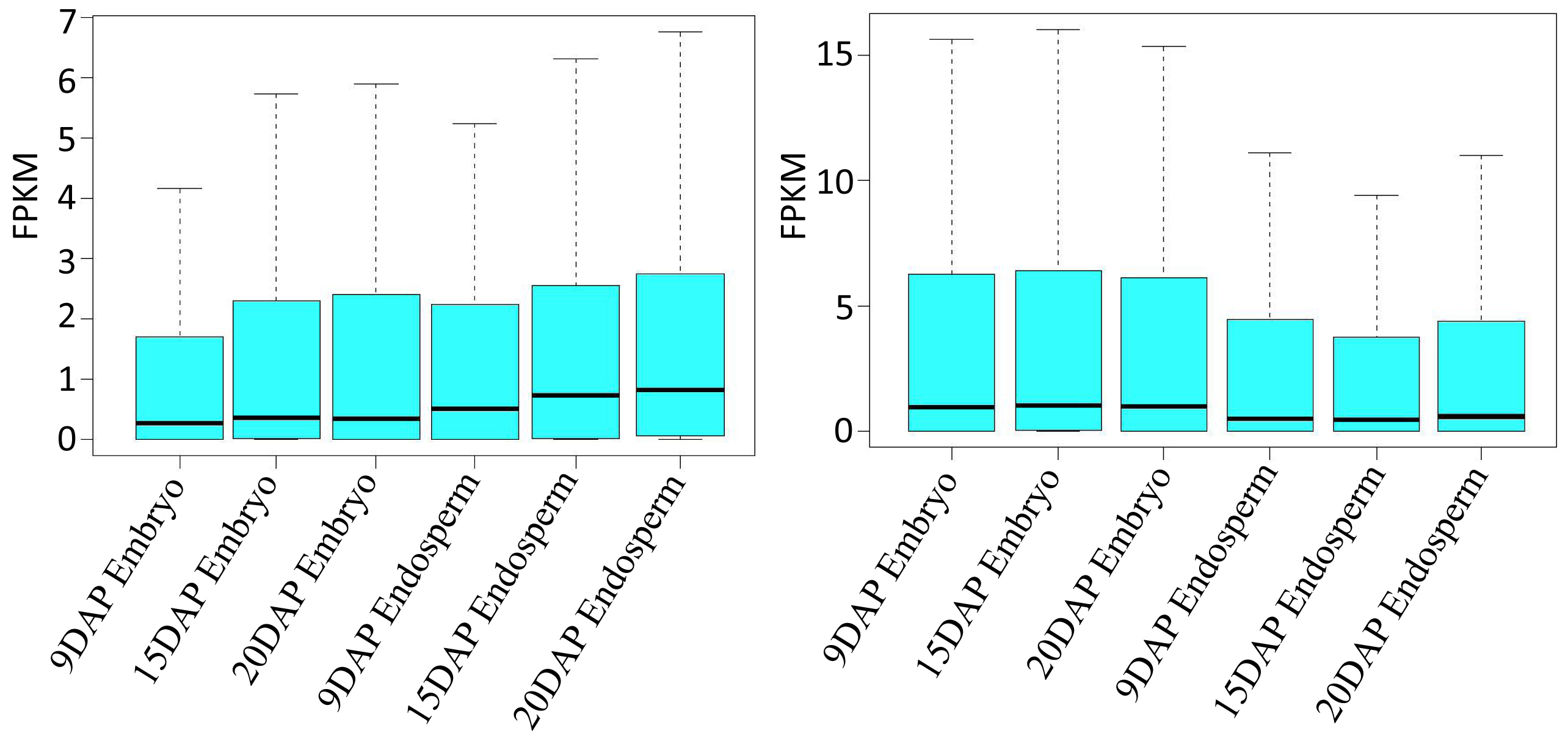

Supplement: Supplementary file 1 [file genes-08-00274-s001.zip › Supplementary files/Figure S2 Expression of lncRNAs and mRNA transcripts.jpg]

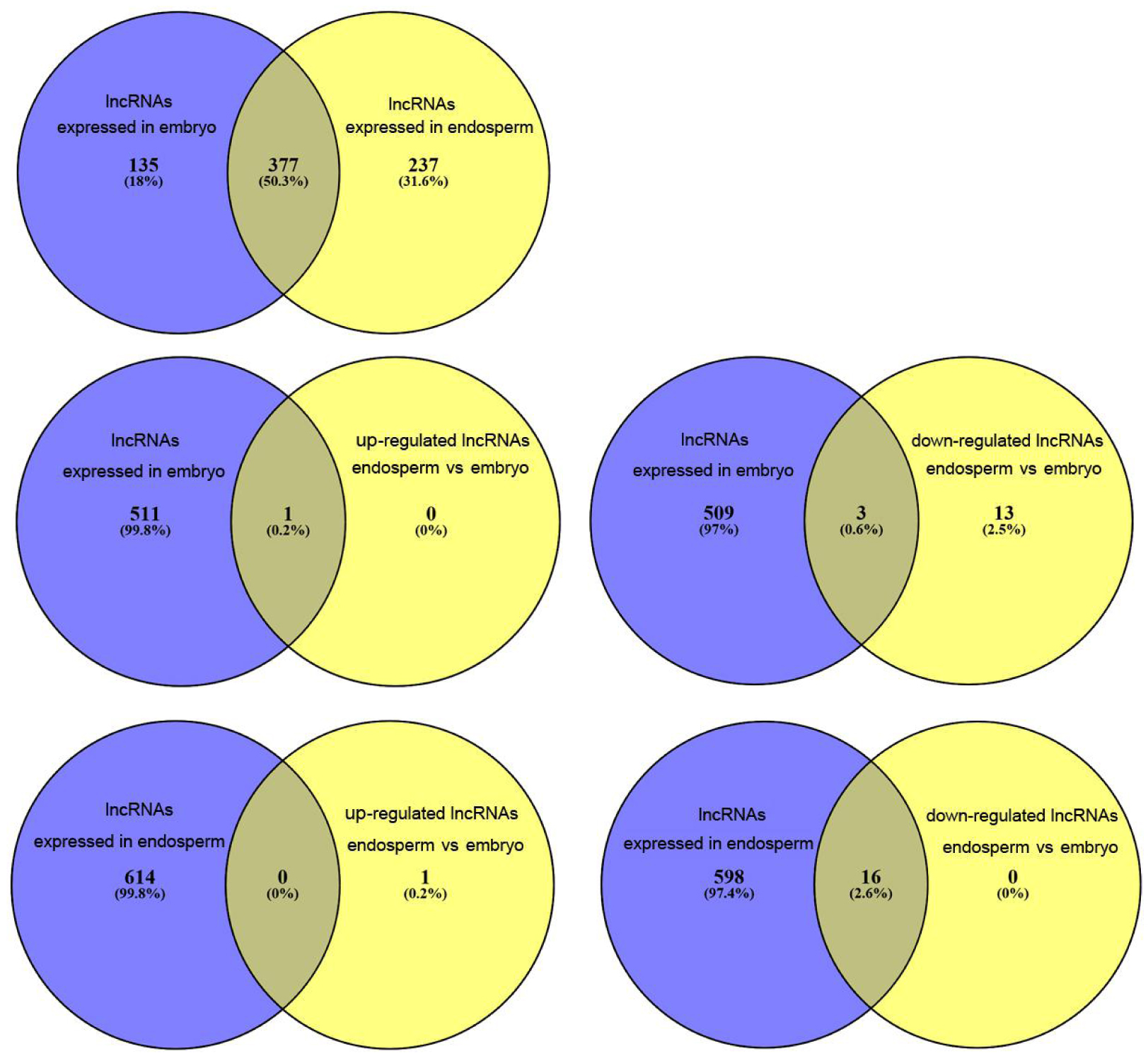

Supplement: Supplementary file 1 [file genes-08-00274-s001.zip › Supplementary files/Figure S3 Expression patterns of embryo or endosperm specific LncRNAs.jpg]
